# Melanin and Melanin-Like Hybrid Materials in Regenerative Medicine

**DOI:** 10.3390/nano10081518

**Published:** 2020-08-03

**Authors:** Chiara Cavallini, Giuseppe Vitiello, Barbara Adinolfi, Brigida Silvestri, Paolo Armanetti, Paola Manini, Alessandro Pezzella, Marco d’Ischia, Giuseppina Luciani, Luca Menichetti

**Affiliations:** 1 Institute of Clinical Physiology, National Research Council, via Giuseppe Moruzzi 1, 56124 Pisa, Italy; paolo.armanetti@ifc.cnr.it (P.A.); luca.menichetti@ifc.cnr.it (L.M.); 2 Department of Chemical, Materials and Production Engineering (DICMaPI), University of Naples Federico II, Piazzale V. Tecchio 80, 80125 Napoli, Italy; giuseppe.vitiello@unina.it (G.V.); brigida.silvestri@unina.it (B.S.); 3 Institute of Applied Physics “Nello Carrara”, National Research Council, via Madonna del Piano 10, 50019 Sesto Fiorentino, FI, Italy; b.adinolfi@ifac.cnr.it; 4 Department of Chemical Sciences, University of Naples Federico II, via Cintia 21, I-80126 Napoli, Italy; paola.manini@unina.it (P.M.); alessandro.pezzella@unina.it (A.P.); marco.dischia@unina.it (M.d.)

**Keywords:** melanin, polydopamine, eumelanin, melanin-like materials, melanin hybrids, regenerative medicine, wound healing, bone tissue engineering, neural tissue engineering

## Abstract

Melanins are a group of dark insoluble pigments found widespread in nature. In mammals, the brown-black eumelanins and the reddish-yellow pheomelanins are the main determinants of skin, hair, and eye pigmentation and play a significant role in photoprotection as well as in many biological functions ensuring homeostasis. Due to their broad-spectrum light absorption, radical scavenging, electric conductivity, and paramagnetic behavior, eumelanins are widely studied in the biomedical field. The continuing advancements in the development of biomimetic design strategies offer novel opportunities toward specifically engineered multifunctional biomaterials for regenerative medicine. Melanin and melanin-like coatings have been shown to increase cell attachment and proliferation on different substrates and to promote and ameliorate skin, bone, and nerve defect healing in several in vivo models. Herein, the state of the art and future perspectives of melanins as promising bioinspired platforms for natural regeneration processes are highlighted and discussed.

## 1. Introduction

**Figure 1:**
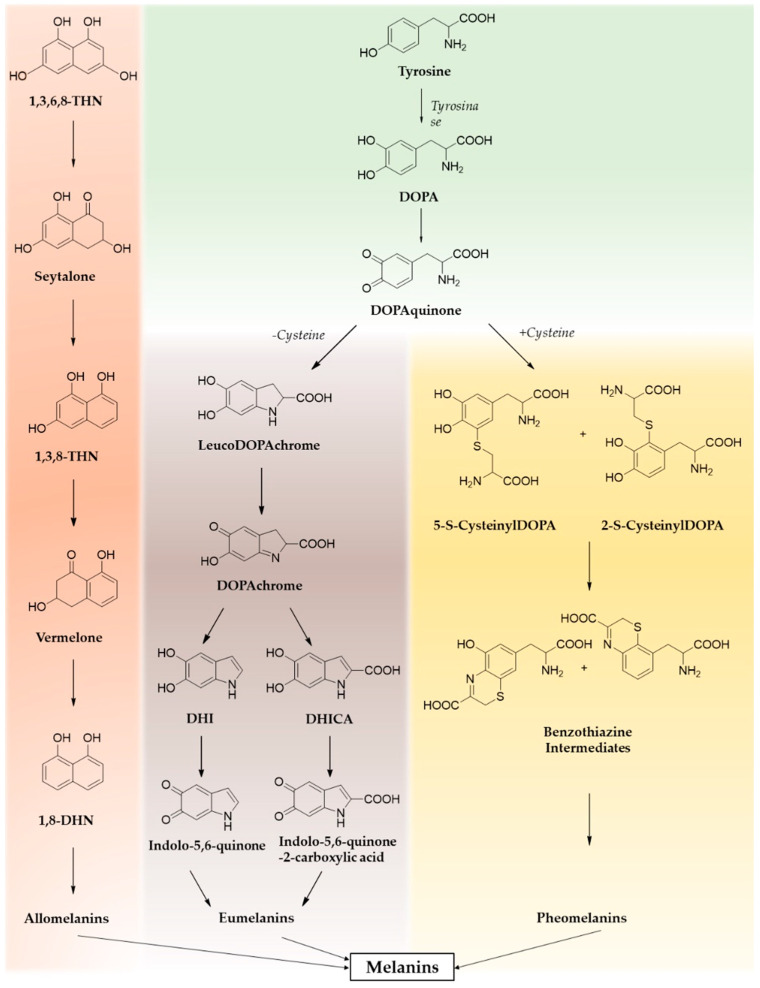
Melanins’ biosynthetic pathways.

The term melanin identifies a heterogeneous group of phenolic polymers found at all levels of the evolutionary scale from fungi and bacteria to plants, mollusks, fish, birds, and mammals, up to man [1]. In mammals, melanin pigments are produced by specialized cells termed melanocytes in the form of granules within cellular organelles known as melanosomes and are responsible for skin, hair, and eye pigmentation, playing a central role in the protective mechanisms against stress-related DNA damage [2,3]. The dark eumelanins originate from the oxidation of the amino acid L-tyrosine to dopaquinone followed by cyclization to 5,6-dihydroxyindole intermediates 5,6-dihydroxyindole (DHI) and 5,6-dihydroxyindole-2-carboxylic acid (DHICA), which eventually polymerize to give the final insoluble pigment [1,4]. Loss of function mutations at the mc1r gene correlates with the red hair phenotype, with a high ultraviolet (UV)-sensitivity and susceptibility to melanoma due to defective epidermal melanization and suboptimal DNA repair [5]. Under these conditions, the eumelanin pathway is impaired; thus, skin pigmentation is dominated by the reddish pheomelanins. These pigments are composed of benzothiazine and benzothiazole units via coupling of dopaquinone with L-cysteine or glutathione [4,6]. Other melanins include neuromelanins, produced in the substantia nigra pars compacta (SNpc) and locus coeruleus, which derive from the oxidation of catecholamines [7,8], allomelanins, and pyomelanins, typical of plants, fungi, and bacteria [9,10]. A schematic representation of melanins’ biosynthetic pathways is reported in Figure 1.

Over the past two decades, melanin pigments, especially eumelanin, have raised a growing interest in the materials science community because of their unique physical and chemical properties, including a broadband absorption spectrum spanning the UV and visible regions [11,12] with a shallow radiative conversion of absorbed photon energy [11,13]. Moreover, eumelanin is characterized by the presence of free phenolic groups that confer to the polymer scavenging ability toward multiple reactive oxygen and nitrogen species (RONS) [14,15]. Together, these properties make eumelanin a good photoprotectant [16]. Depending on its hydration state, eumelanin also displays pronounced electrical conductivity [17] and paramagnetic behavior [9,18]. These electronic properties may explain the presence of eumelanin in electrically active tissues such as SNpc. Furthermore, due to the abundance of binding groups (amine and catechol among others), eumelanins display a chelating capacity [19], which has been suggested to prevent dopaminergic neurons from accumulating toxic ions, such as iron which are involved in degeneration and death [20,21]. The aforementioned properties make eumelanin a promising platform for several biomedical applications, including tissue regeneration, bioelectronics, drug-delivery systems, antioxidant therapy, and multimodal imaging.

This review highlights recent advances as well as challenges in the design of melanin-based materials as bioinspired platforms to support and promote natural regenerative processes. First, synthesis approaches of melanin-like materials will be outlined with a particular focus on hybrid systems obtained by melanin combination with inorganic and/or organic components. Furthermore, the most challenging issues of biomimetic processes to melanin formation will be outlined and discussed. Then, the most intriguing physical–chemical properties of melanin-like materials for biomedical applications will be highlighted. Finally, the most promising applications and perspectives of melanin-like materials in regenerative medicine will be analyzed. This study is expected to provide strategic guidelines for the development of cutting-edge melanin-based materials for regenerative medicine.

## 2. Synthetic Melanin-Like Materials: Opportunities and Issues of Biomimetic Approaches

Natural melanins are usually available as solid granules deeply embedded in their biological matrix that are difficult to isolate without significant alteration during extraction and purification treatments [22]. These issues limit the practical utility of natural melanins [23] and urge the quest for valuable and more accessible alternatives.

Synthetic analogs of natural melanins can be produced through in vitro routes based on the oxidation of melanogenic precursors and constitute reliable models to unveil functions and structure–properties relationship of these pigments [23,24,25]. The oxidation methods which can be applied include self-oxidation in alkaline environment, oxidation by ferrocyanide compounds, or biomimetic enzymatic oxidation [26]. Stronger oxidation agents, including ammonium persulfate, sodium periodate, copper sulfate [27,28], and light irradiation [29], have also been explored, improving overall polymerization efficiency. However, harsh synthesis conditions cannot be applied to systems containing cell cultures.

Practically, any moieties in the melanogenic pathway and even natural phenolic amines [30] can be employed as starting precursors, most frequently tyrosine, l-3,4-dihydroxyphenylalanine (L-DOPA), DHI, and DHICA. Dopamine (DA) has been the focus of considerable interest, too. DA has been exploited as a precursor for a biocompatible melanin-type polymer termed polydopamine (PDA) widely used for surface functionalization. Inspired by the adhesive properties of mussels, in 2007, the Messersmith group described the efficient adhesion properties of the species produced by autoxidation of DA at pH 8.5 [31] on a wide variety of materials, including organic and inorganic surfaces, providing a smart platform for secondary functionalization technologies [32]. Notably, PDA structural properties can be tailored on specific application requirements by varying the synthetic conditions (i.e., DA concentration or buffer composition) [33]. Recently, norepinephrine (also known as noradrenaline), a neurotransmitter and vasopressor moiety, widely employed in medicine, has been proposed as a novel melanogenic precursor [34,35]. It undergoes oxidative polymerization forming both monodisperse nanoparticles [34] and thin coatings, with tough adhesive properties on different substrates.

Highly promising strategies toward the development of effective strategies to increase the chemical stability and to fine-tune the physicochemical properties of melanin biopolymers by rational control of π-electron conjugation have recently been inspired by the superior paramagnetic properties of synthetic fungal melanin (mycomelanin) mimics. Poly-1,8-dihydroxynaphthalene (pDHN) displays a higher degree of structural integrity compared to typical synthetic eumelanins and a strong radical scavenging capacity associated with an intense electron paramagnetic resonance signal (g = 2.0030). Morphological data indicated amorphous aggregates of small globular structures with an estimated stacking distance of 3.9 Å and broadband absorption in the visible range [36]. Thin films from pDHN were found to display high structural regularity, an ultrasmooth morphology, with excellent robustness against peroxidative bleaching and good adhesion under watery conditions, good biocompatibility, and remarkable effects in promoting differentiation of embryonic stem cells prevalently towards the endodermal lineages without additives [37].

Both the structural and physicochemical properties and the supramolecular features of synthetic melanins are susceptible to the synthetic protocols and the conditions used for polymerization [23,26]. As an alternative to the conventional solution oxidation methods, electropolymerization has also been employed as a simple and efficient route for PDA synthesis; however, it only allows to surface deposition on conductive materials, this restricts its application [38]. In any case, providing stable nanoparticles in aqueous or biological media is a challenging task due to the many concurrent factors controlling polymerization and self-assembly. The use of suitable additives capable of modulating the manifold competing factors during the polymerization/self-assembly process can address this issue. Employed species include surfactants, polyelectrolytes, ionic liquids proteins, and other organic compounds. [39,40].

Polymers, such as polyvinyl alcohol (PVA), can control DA polymerization leading to stable PDA sols [15]. Recent studies also proved the efficacy of several proteins in controlling the size, morphology, and optical properties of synthetic melanin nanoparticles [41,42]. Alternatively, tris buffer solutions [43], as well as UV-irradiation under acidic or neutral conditions, proved able to limit particle growth [44].

**Figure 2:**
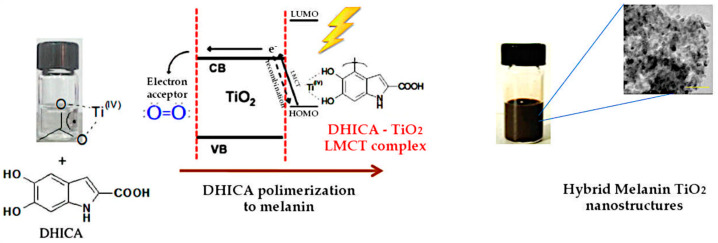
Photocatalytic activity of TiO_2_ allows for 5,6-dihydroxyindole-2-carboxylic acid (DHICA) polymerization and formation of melanin–TiO_2_ hybrid nanostructures with unique biocide activity. Reproduced with permission from [47], and from [45]. Copyright American Chemical Society, 2016 and The Royal Society of Chemistry, 2015, respectively.

Recently, following a bioinspired approach, biocompatible-nanostructured ceramic phases were exploited as catalysts and structure-directing agents during melanin formation, thus mimicking melanosomes [22,45,46]. The ceramic-templated approach appears as a promising eco-friendly strategy in the field of melanin-like materials, leading to monodisperse and biocompatible melanin-based hybrid nanoparticles and able to boost melanin’s intrinsic properties, such as antimicrobial and antioxidant action (Figure 2) [22,45,46].

### 2.1. From Physicochemical to Biomedical Properties of Melanins and Melanin-Like Hybrid Materials

#### 2.1.1. Versatile Chemistry and Easy Chemical Coupling

Natural and synthetic melanins share the abundance of functional groups in their molecular backbone [42]. Carboxyl (−COOH), aromatic amine (−NH), hydroxyl (−OH), as well as catechol groups, can act as binding sites to allow easy functionalization with a considerable range of biologically active moieties. Moreover, carboxyl and amino groups can be involved in amidic bond formation through 1-ethyl-3-(3-dimethylaminopropyl)carbodiimide (EDC) chemistry [42,48]. Furthermore, nucleophilic thiol- and amino-containing moieties can be easily grafted by several coupling reactions [49]. Moreover, the presence of a vast set of surface functional groups makes melanin easily combinable with different surfaces such as metal oxides and ceramics.

Recently, melanin-based hybrid systems have been improved with inorganic silica coatings to enhance biocompatibility, surface functionality, and aqueous dispersity for biomedical applications. For example, Gadolinium-chelated melanin nanoparticles were successfully synthesized and covered with a silica shell [50]. The silica nanocoating significantly improved the magnetic resonance r1 relaxivity of the systems. In addition, they demonstrated high heat transduction efficiency coupled with sufficient tumoricidal heating, allowing their use as in vivo dual-modal magnetic resonance imaging (MRI)/fluorescent imaging nanoplatforms. Furthermore, nanostructured silica was also employed as a templating agent for the eumelanin phase, tuning its supramolecular structure [51]. One-pot in situ synthesis strategy was carried out, using silica, DHICA, and Ag as starting precursors. This strategy allowed self-structuring of the system into a core–shell architecture, where the Ag core was found to show stable photoacoustic properties even under prolonged irradiation. Hybrid functional nanoarchitectures were fabricated, through a hydrothermal synthesis, integrating a eumelanin-like polymer with TiO_2_ via LMCT (ligand to metal charge transfer)-based photooxidative process [22,45,46].

#### 2.1.2. Metal Ions Chelating Action

**Figure 3:**
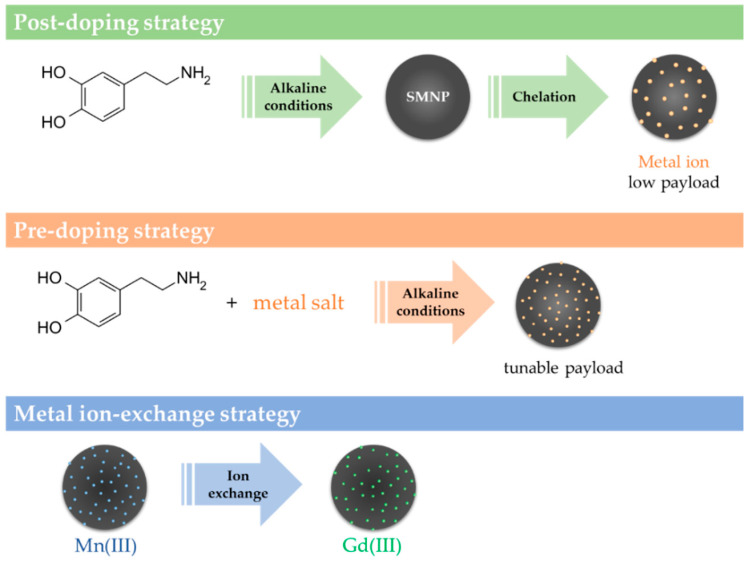
Schematic of the strategies for preparing metal ion-loaded melanin nanoparticles. SMNP synthetic melanin nanoparticles. Reproduced with permission from [52]. Copyright Elsevier, 2020.

The abundance of metal ions coordination sites in melanins’ structure results in a high affinity for metal cations. This attitude has been extensively exploited to bind different metal ions to improve melanin’s intrinsic features, such as antioxidant and light absorption performance [52,53], but also to confer further non-native contrast properties [48,49,52]. Loading strategies include postdoping, predoping, and metal ion-exchange approaches (Figure 3). Postdoping can be applied even to natural melanins and allows to control properties of melanin nanoparticles, yet only low payloads can be achieved. The predoping approach addresses this limitation and enables tunable metal ions amounts within nanoparticles [54]. The ion-exchange strategy is usually exploited when direct doping cannot be achieved due to aggregates formation [52].

**Figure 4:**
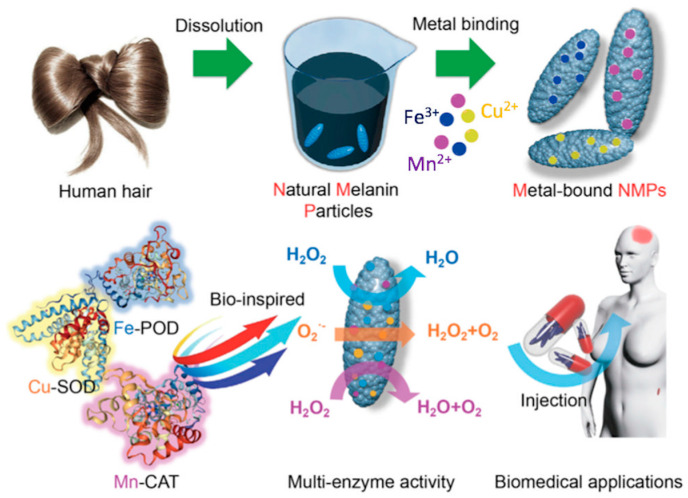
Enzyme mimicking activity of metal ions-doped melanins for various biomedical applications. Reproduced with permission from [53]. Copyright Cell Press, 2020.

Recently, hair-extracted melanin nanoparticles doped with metal ions demonstrated the ability to mimic natural enzyme activities, revealing potent anti-inflammatory and antibacterial activity, thus opening new scenarios in the design of biomedical materials (Figure 4) [53]. Metal ions doping enhances significantly light absorption, as well as the photothermal conversion of melanins [52]. Furthermore, melanin complexation with different metal ions makes them a versatile probe for multimodal imaging, combining different imaging methods with peculiar resolution and penetration depth [55,56,57]. Moreover, metal complexes of melanogenic precursors play a crucial role in driving the steps of the melanogenic pathway, ultimately controlling melanin’s biological functions. As a proof of concept, Ti(IV) complexes with different melanogenic precursors determine the fate of obtained hybrid nanostructures as either antimicrobial or good antioxidant agents [22].

#### 2.1.3. Broad Light Absorption

**Figure 5:**
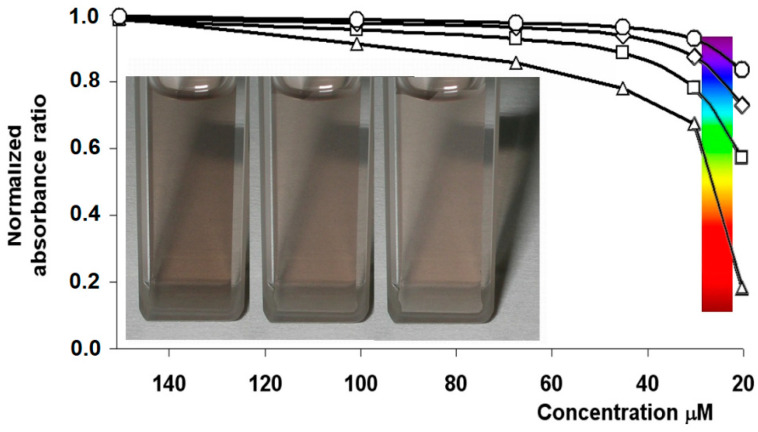
Normalized plots of the absorbance ratios Ax/A314 at selected wavelengths (○ 384 nm, ◇ 420 nm, □ 550 nm, and △ 750 nm) as a function of dilution of soluble eumelanin (for all wavelengths, normalization was obtained by dividing all absorbance ratios by the relevant value at 150 mM concentration).

One primary biological function of melanins is photoprotection and screening against U.V. harmful light [58]. The brown-black color of eumelanins is direct evidence of their efficient light absorbance behavior, covering a wide range of UV–Visible spectrum as a consequence of contribution from both intrinsic and extrinsic chromophores (Figure 5) [59].

Melanin-like materials keep these optical features; additionally, close control of particle size allows color tailoring and skin-matching of produced nanoparticles, which are easily internalized into keratinocytes, protecting the cells from UV damage [42,60]. Furthermore, these pigments are extremely efficient in converting light energy into heat [49,61], holding huge potential as active components in photothermal therapy (PTT) against cancer [62,63] but also against drug-resistant pathogens [64].

**Figure 6:**
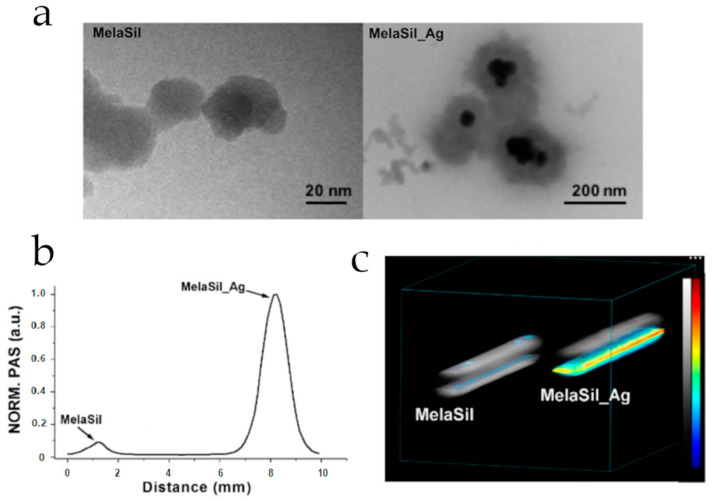
Transmission electron microscopy (TEM) of both melanin-silica (MelaSil) and Ag-melanin-silica hybrid (MelaSil_Ag) photoacoustic nanoprobes (**a**) Comparison of normalized mean photoacoustic signal (**b**) and 3D photoacoustic-ultrasound render (**c**) produced by nanoparticles with and without Ag components; in grayscale, the intensity of the ultrasound signal, whereas in colored scale, intensity of photoacoustic signal. Reproduced with permission from [51] Copyright Elsevier, 2019.

The photoactivity of melanins has been exploited to realize a large variety of hybrid materials for biomedical applications. Recently, the huge potential of eumelanins as photoacoustic probes was realized using a multicomponent system where a ceramic phase (SiO_2_) acted as a templating agent for eumelanin, which, in turn, produced Ag nanoclusters formation, through its chelating and reducing properties (Figure 6) [51].

Furthermore, melanins have been used as doping agents to increase the UV response of PVA, one of the most common biopolymers used in medical applications [65], obtaining a new hybrid biomaterial with unique properties for optical applications. Similarly, networks that are composed of well-characterized synthetic polymers and natural melanin pigments found within the human body have been recently proposed. Melanin nanoparticles have been chosen for doping of photodegradable self-assembled hydrogel networks of poly(L-lactide-co-glycolide)–poly(ethylene glycol) (PLG–PEG) ABA triblock copolymers to produce reconfigurable networks based on photothermal phase transitions, representing a potential strategy for photodegradable polymers with increased likelihood for clinical translation [66]. On the other hand, melanins can absorb near-infrared (NIR) light with a high photothermal conversion efficiency. This photothermal effect has been used for the development of new drug release systems to control drug delivery in a specific region. Recently, hybrid melanin-alginate microparticles have been produced, showing int+eresting properties to locally irradiate target region, guaranteeing high biocompatibility and low toxicity [67]. Similarly, nanovesicles composed of phospholipids incorporating melanin, poly(N-isopropylacrylamide-co-acrylamide) (PNIPAM), and 5-fluorouracil (5-FU) has been proposed for thermoresponsive drug release by NIR laser irradiation, minimizing side effects and facilitating a rapid drug release at the lesion site [68].

**Figure 7:**
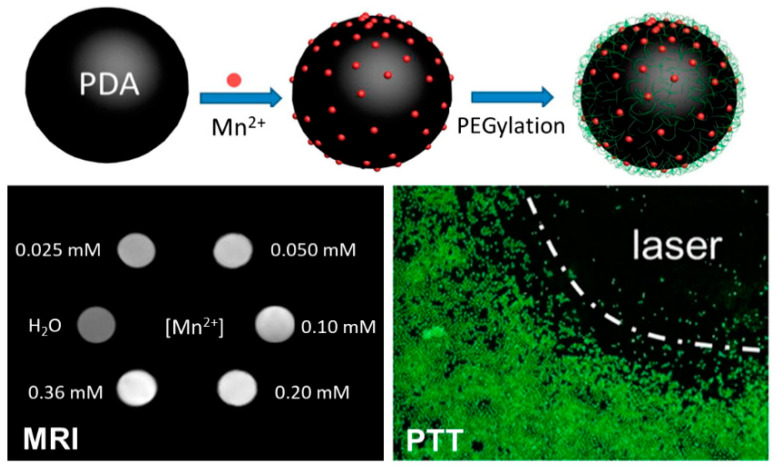
Schematic illustration of the fabrication of PEGylated Mn^2+^−PDA nanoparticles (PMPDA NPs). MRI in vitro assay of PMPDA NPs: T1-weighed images of water and PMPDA NPs in aqueous solution at different concentrations of Mn^2+^ ions and fluorescence microscopy images of HeLa cells stained with calcein AM after treatment with both PMPDA NPs and 10 min of laser irradiation. Reproduced with permission from [69]. Copyright American Chemical Society, 2015.

Today, a considerable variety of melanin-like hybrid nanostructures were proposed by either covalent cross-linking or physically entrapping, during synthesis step, inorganic components or metals. Among them, considerable efforts have been spent on the development of nanoparticles-based systems to integrate, into one entity, multiple functional components. The intrinsic chelating properties of melanin and melanin-like materials were exploited to design novel theranostic agents [56,69,70]. For example, PDA has been exploited to chelate Mn^2+^ ions. The resulting hybrid nanoparticles showed a significant MRI signal enhancement, low toxicity, and good photothermal activity (Figure 7) [69].

An alternative bioinspired strategy was applied to chelate Fe^3+^ ions [70]. The synthesized nanoparticles showed extended adsorption in the NIR region and significant enhancement in MRI signal, resulting in an excellent biocompatible smart theranostic nanostructure. Following similar “chelator free” strategy, PEGylated melanin nanoparticles were also functionalized with Gd^3+^ and ^64^Cu^2+^ ions [56], to exploit the potentiality of the system for multimodality imaging, including positron emission tomography (PET), MRI, and photoacoustic imaging (PAI).

**Figure 8:**
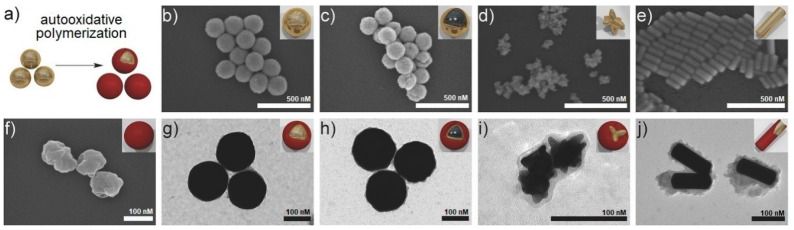
Synthesis of melanin-coated gold particles. (**a**) Auto-oxidation of dopamine to melanin can be conducted as a seeded dispersion polymerization to deliver gold-core melanin-shell particles. (**b**–**f**) Scanning electron micrographs (SEM) of pristine and melanin-coated gold nanoparticles: (**b**) spherical gold particles (Au), (**c**) spherical silica-core gold-shell particles (SiAu), (**d**) gold nanostars (AuStars), and (**e**) gold nanorods (AuRods). In (**b**–**e**), the scale bars represent 500 nm. (**f**) SEM image of melanin particles without a gold core. (**g**–**j**) TEM images of melanin-coated (**g**) spherical gold nanoparticles (AuMel), (**h**) silica-core gold-shell particles (SiAuMel), (**i**) gold nanostars (AuStarsMel), and (**j**) gold nanorods (AuRodsMel). In (**f**–**j**), the scale bars represent 100 nm. The insets display an artistic rendering of the individual particle geometries. Reproduced with permission from [71]. Copyright John Wiley and Sons, 2018.

The coordinative binding capability of the catechol unit of melanin-like components was also exploited to enable the formation of melanin shell around different types of metal nanoparticles. Gold-core melanin shell nanoparticles with different geometries (from spheres to nanostars and nanorods) were quickly produced through auto-oxidative polymerization of DA in the presence of gold nanosystems (Figure 8) [71]. The absorption bands of nanoparticles could be tuned by varying the dimensions and geometry of the particles, and at the same time, the surface functionalization of the melanin coating increased biocompatibility of gold nanoparticles (GNPs).

**Figure 9:**
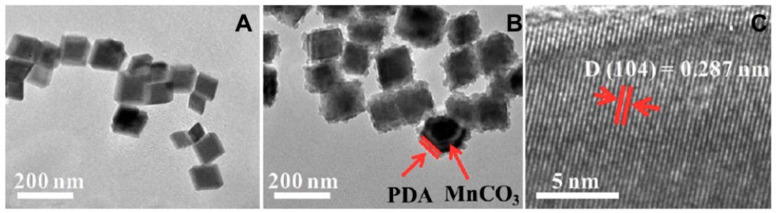
Characterization of the MnCO_3_ and MnCO_3_@PDA NPs: (**A**) TEM image of MnCO_3_, (**B**) TEM image of MnCO_3_@PDA, and (**C**) HRTEM image. Reproduced with permission from [73]. Copyright American Chemical Society, 2017.

Additionally, melanin could be coated on the surface of different types of template-core material. For example, melanin-mediated biomineralization method was exploited to prepare different metal carbonates nanoparticles. Following this synthetic way, calcium carbonate-PDA hollow nanoparticles were simply synthesized [72]. The choice of endogenous components resulted in highly biocompatible systems with no long-term side effects. These features make them ideal candidates as multimodal imaging nanoplatforms. Following a similar methodology, manganese carbonate-PDA core–shell nanocomposites were also produced as potential MRI/PTT theranostic agents (Figure 9) [73].

#### 2.1.4. Paramagnetic and Red-Ox Properties

Melanins possess intrinsic paramagnetic properties due to the complex interplay of interacting catechol-quinone moieties favoring p-electron delocalization and transfer both intramolecularly and intermolecularly. In addition, they show tunable redox behavior, which confers them the role of free radical scavengers, being able to react with RONS, thus mitigating oxidative stress underlying many chronic degenerative pathologies. Their antioxidant function is particularly relevant in biomedical applications [32,48,49]. Melanin-like materials with improved antioxidant activity have been obtained by conjugating natural or synthetic melanins with other organic components, such as polymers or biopolymers. For example, melanin nanoparticles isolated from the Sepia ink have been used as functional fillers for the preparation of agar-based functional films, which showed a high antioxidant activity comparable to ascorbic acid [74]. Similarly, synthetic melanin-like nanoparticles, have been conjugated with chitosan (CS), producing nanocomposite films with strong antioxidant activity [75]. Interestingly, a water-soluble humic acid and melanin-like polymer complex has been isolated from olive mill wastewaters, showing a high scavenging activity [76].

In the green synthesis of organic/inorganic hybrid nanoparticles, eumelanins redox properties were extensively investigated. The presence of catechol and N-H functional groups endowed eumelanins with an active anchoring site, and neither surface modification nor additional reductants were needed for absorbing and reducing metal ions [77,78]. Silver nanoparticles were also uniformly distributed on the surface of PDA nanoparticles to produce hybrid materials. The eumelanin component effectively prevented silver nanoparticles aggregation during the synthesis, avoiding, at the same time, possible release of the ions [78], leading to hybrid material with high antibacterial activity.

#### 2.1.5. Adhesive Properties

Eumelanin adhesion on a great variety of surfaces, including organic as well as inorganic materials, observed after the polymerization of suitable precursors, is carried out while interfacing at the desired surfaces, namely, solid-phase DHI polymerization over glass or quartz substrates results in the formation of adhesive thin films also capable of resisting immersion in cell culture media over weeks [79].

Melanins’ adhesion can be obtained either by polymerization of suitable precursors like dopamine or by the addition of long-chain diamines during the polymerization of catechol compounds [80,81].

**Figure 10:**
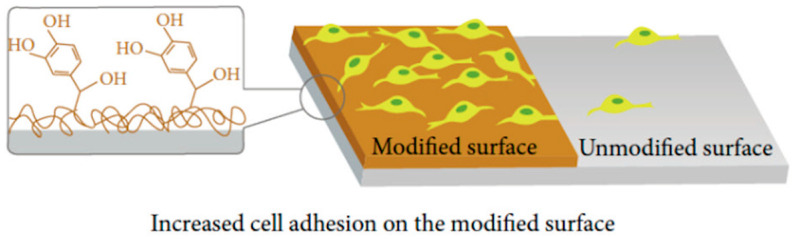
Surface modification through melanin-like coatings obtained by polymerization of norepinephrine promotes adhesion of human stem cells on obtained biointerfaces. Reproduced with permission from [35]. Copyright Hindawi Publishing Corporation, 2014.

The adhesion properties of DHI-based melanins also allowed a variety of surface functionalizations aimed to fabricate bioactive substrate for stem cell culture and differentiation [82,83]. In particular, eumelanin-coated poly(lactic acid) (PLA) microfibers proved to be capable of supporting survival, adhesion, and differentiation toward a more mature neuronal phenotype of neuroblastoma cell type, also opening to applications in bioelectronics [84]. These features have also been exploited to achieve immobilization of biomolecules on different surfaces. To this purpose, both in situ and ex post strategies can be carried out, i.e., in the former, biomolecules are dissolved with the melanogenic precursor during its polymerization, whereas in the latter approach, immobilization is achieved through different chemistry approaches after polymerization [85]. Melanin-like materials play a crucial role in instructing cellular surface distribution [60]. Thus, controlling the spatial organization of surface coatings can result in the patterning of cell differentiation, proliferation, and migration (Figure 10) [35].

Hybrid melanin-like materials with improved adhesive properties can be obtained by a combination of melanins with synthetic polymers. For example, DA was used to modify polyaniline (PANI) via a one-step chemical oxidization method. Compared to pure PANI, the modified PANI exhibited greatly enhanced adhesion force to the substrate, improved biocompatibility, and hydrophobicity, also maintaining good electrical conductivity [86]. These properties have proposed them as a promising surface coating of implant materials or conductive platforms in tissue engineering.

Table 1 summarizes the main hybrid melanin-like materials and their properties exploited for the application they are designed for.

## 3. Melanins and Melanin-Like Materials in Tissue Engineering

### 3.1. Wound Healing

An ideal wound dressing should possess multiple properties. First, it should provide a moist environment to prevent dehydration and favor angiogenesis and re-epithelialization [89]. In addition, it should absorb exudates, allow gas permeation, and provide protection against bacteria and other external factors [90]. Newer and improved dressings should show superior characteristics, including controlled biomacromolecule (growth factors, cytokines, and proteins) or drug delivery, antioxidative properties, self-healing ability, and conductivity [91]. Melanins meet many of such requirements. Consequently, over the last few years, many melanin-doped dressings to address impaired wound healing have been established. Melanin and melanin-like polymers have been incorporated into hydrogels, fibrous membranes, films, and other matrixes in order to improve the physical, chemical, and mechanical characteristics of the matrixes themselves, as well as to deliver therapeutic agents. The following sections offer an overview of recent applications of melanin-modified materials in wound healing.

#### 3.1.1. Hydrogels

**Figure 11:**
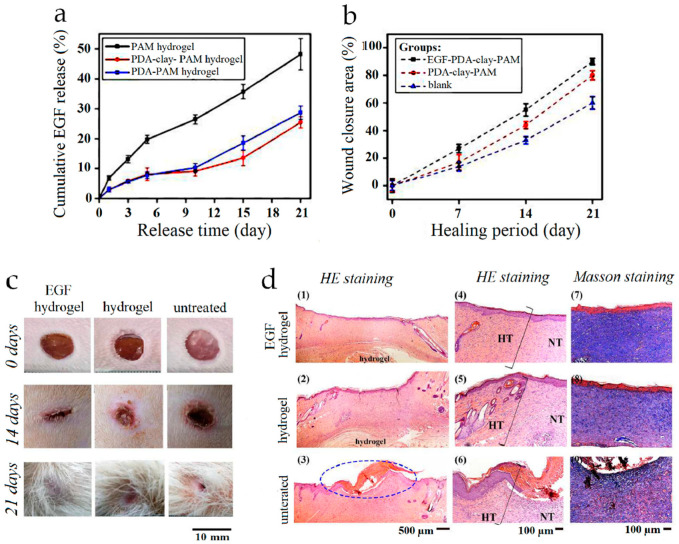
Polydopamine (PDA)-clay-polyacrylamide (PAM) hydrogel as a wound dressing in a full-thickness skin defect. (**a**) Cumulative EGF released from PDA-clay-PAM, PDA-PAM, and clay-PAM hydrogels in PBS. (**b**) Wound closure of untreated defects and defects treated with EGF-loaded PDA-clay-PAM, EGF-free PDA-clay-PAM, and PDA-PAM. (**c**) Digital photos of the wound after 0, 14, and 21 days of healing. (**d**) Photomicrographs showing histological staining of wound sites on day 21. (1−3) Overview of the defects treated by epidermal growth factor (EGF)-loaded PDA-clay-PAM hydrogel and PDA-clay-PAM hydrogel and untreated defect. (4−6) The interface between the newly regenerated tissue (NT) and host skin tissue (HT). (7−9) Masson staining of the collagen deposited in the defects. Reproduced with permission from [94]. Copyright American Chemical Society, 2017.

The potential of hydrogels for the treatment of skin wounds has been extensively explored in the last few years [91]. Besides the ability to absorb exudates, isolate the wound, and mimic the three-dimensional structure of tissues, their cross-linked structure enables the incorporation of different bioactive agents [91,92]. In addition, hydrogels can be injected directly on the wound, matching any shape of the defect site. Nonetheless, they often present poor adhesiveness and mechanical properties, which can cause damages or ruptures, thus affecting the dressing performance [93]. Surface modification of hydrogels with natural melanin or melanin-like polymers has been widely reported to improve biocompatibility, mechanical properties, and adhesiveness to the wound. Han and colleagues have proposed an epidermal growth factor (EGF)-doped polyacrylamide (PAM) hydrogel containing PDA-intercalated clay nanosheets [94]. The resulting material demonstrated higher mechanical properties due to the interaction between PDA and the clay-hydrogel network and excellent adhesive strength to several materials, including human skin. Such adhesive properties were maintained over time, allowing repeated use and prolonged storage of the material. In addition, in vitro and in vivo experiments indicated that PDA promoted cell adhesion and proliferation and enhanced wound healing in a rat skin defect model (Figure 11).

PDA has also allowed to overcome the poor hydrophilicity and bioavailability of potential therapeutic agents and improve their release profile. For instance, PDA nanoparticles have been used as a carrier for the antioxidant molecule puerarin (PUE) in a polyethylene glycol-diacrylate (PEG-DAc) hydrogel [95]. Compared to the bare PUE formulation, PDA/PUE hydrogel showed a better drug release profile, enabling the sustained delivery of the antioxidant drug. Moreover, the authors described an excellent cytocompatibility, while in vivo results showed several positive effects, including protection against infections, acceleration of healing, and amelioration of the aesthetical appearance of the wound.

Due to its highly reactive feature, PDA has also been observed to endow different materials with self-healing properties. To this end, several studies have recently reported PDA-modified materials as self-healing wound dressings [93,96,97,98]. This ability allows the hydrogels to repair themselves in case of damage, extending their durability and improving their capability to prevent infections.

Besides its successful application as a surface modification agent, melanin has been investigated as a treatment itself to promote tissue regeneration. Da Silva et al. have explored the effect of eumelanin nanoparticles extracted from *Sepia officinalis* on human keratinocytes (hKCs) [99]. In vitro results demonstrated that hKCs internalized eumelanin nanoparticles without cytotoxic effects; moreover, hKCs pretreated with eumelanin nanoparticles showed a marked decrease of RONS production following UV-A/UV-B irradiation. The incorporation of eumelanin nanoparticles into a spongy-like hydrogel determined the modification of the physicochemical characteristics of the hydrogel itself, thus permitting the sustained release of eumelanin. Further in vivo experiments proved that the eumelanin hydrogel induced a physiological host response after implantation and that the amount of released eumelanin was in the range of concentrations expected to exert beneficial effects on hKCs. In addition, the authors hypothesized that the conductive and antioxidant properties of eumelanin could sustain the propagation of electric signals and the recruitment of keratinocytes as well as to contribute modulating the inflammatory response at the wound site [99].

As discussed, melanin-modified materials show a prominent photothermal activity that can be exploited for several purposes. NIR light treatment has a great potential to treat infections by generating ROS, which are able to kill pathogenic microorganisms [100]. Based on this, Han et al. have recently developed NIR-responsive chitosan/silk fibroin (CS/SF) cryogels incorporating PDA nanoparticles [101]. By exploiting the PDA-mediated photothermal effect, the authors observed a significant dose-dependent antibacterial activity of the hydrogels following NIR irradiation. Besides, NIR irradiation did not show harmful effects on fibroblasts, while the presence of PDA positively affected cell adhesion, proliferation, and morphology. Data obtained in full-thickness skin defect experiments demonstrated that PDA-CS/SF cryogels significantly increased the wound healing rate compared to CS/SF alone; additionally, the exposure of the wounds to NIR light induced a faster healing rate and better tissue regeneration.

The antibacterial activity of hydrogel dressings can be further enhanced, combining a PDA-mediated photothermal treatment with antibiotic drugs. Liang et al. have developed some polymeric composite hydrogels incorporating PDA and doxycycline [97,102]. The composite PDA hydrogels demonstrated photothermal-mediated antimicrobial activity and sustained antibiotic release capacity. Moreover, both the formulations demonstrated hemostatic, tissue-adhesive, antioxidant, and conductive properties. Electric signals induce the expression of regenerative-related factors and promote cell migration at the wound site [103]; thus, melanin-doped conductive wound dressings could represent an innovative strategy to enhance the healing process.

**Figure 12:**
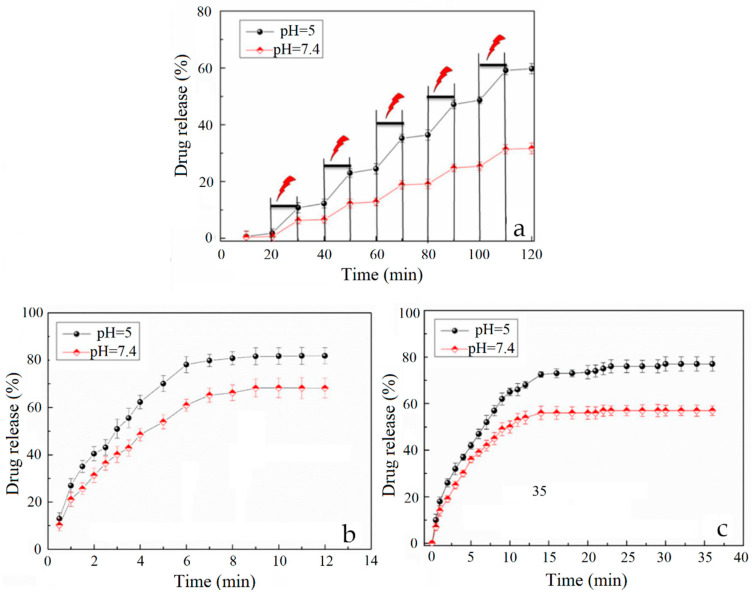
(**a**) NIR light-triggered (2.0 W/cm^2^ for 10 min) drug release from PDA hydrogel in PBS (pH = 5.0 and pH = 7.4) at 37 °C. In vitro drug release profiles of TH from bare hydrogels (**b**) and PDA hydrogels (**c**) over a longer period time. Reproduced with permission from [104]. Copyright Elsevier, 2018.

The photothermal properties of melanins can also be exploited to create on-demand drug release devices. On this path, Liu et al. have proposed a multiresponsive composite hydrogel incorporating PDA and tetracycline hydrochloride (TH) [104]. In vitro results evidenced that the presence of PDA enabled the controlled release of TH under both NIR exposure or low pH conditions (Figure 12). Indeed, on the one hand, the temperature rise induced by NIR irradiation allowed TH release in an “on-off” fashion as a consequence of the swelling/deswelling process of the polymer network. On the other hand, mild acidic conditions caused the progressive disruption of PDA/TH chemical interactions inducing the sustained release of TH. A similar approach was reported by Gao et al., who have designed a ciprofloxacin (Cyp)-loaded PDA NP glycol-chitosan (G-C) hydrogel [105]. Here, NIR irradiation worked as a trigger for the on-demand release of Cyp allowing spatial and temporal control of the antibiotic treatment; moreover, the synergistic effect of heat and Cyp led to an excellent antibacterial efficacy. In addition, Han et al. have integrated PDA nanoparticles into a PNIPAM hydrogel to create a triple-responsive device [96]. By exploiting NIR irradiation, the hydrogel allowed controlled activation, drug release, and healing ability.

**Figure 13:**
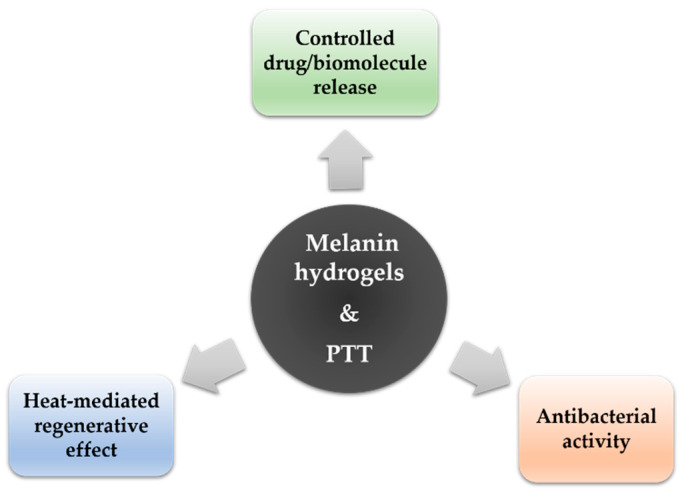
Photothermal-responsive melanin hydrogels: applications in wound healing.

From a different perspective, melanin photothermal properties can be exploited to generate heat and thus allow the photothermal treatment of the wounds. NIR light treatment has been proven effective in accelerating wound closure by modulating the expression of soft tissue regeneration and inflammation-related genes [106]. Moreover, it has been demonstrated that mild heating of the wound site can promote recovery as a result of an increase in blood flow and cell proliferation [107]. With an original approach, Gao et al. have combined PDA and graphene oxide (GO) with PAM to obtain a self-adhesive photothermal hydrogel able to convert solar light into heat efficiently [108]. In vivo experiments under simulated solar light conditions demonstrated that the hydrogel film could effectively accelerate wound closure while providing antibacterial effect. Figure 13 summarizes the application of photothermal-responsive melanin hydrogels.

#### 3.1.2. Films and Membranes

Fibrous membranes, compared to other dressings, presents several attractive structural features, including an extracellular matrix (ECM)-mimicking architecture, high and modulable porosity, and a large area to volume ratio. Moreover, as for the hydrogels, fibrous membranes can be functionalized or loaded with bioactive molecules and drugs. Fibrous biomaterials are uncommon in nature, but several organic polymers are suitable for the production of biodegradable fibrous dressings through different techniques, such as electrospinning, molecular self-assembly, or thermally induced phase separation [109,110].

Silk fibroin (SF) dressings are gaining increasing attention due to their good biocompatibility, processability, and mechanical properties; nonetheless, their wound healing ability needs further implementation. PDA coating has been widely used to improve the healing properties of SF membranes. PDA can be easily deposed on SF membranes by merely soaking the electrospun membranes in a DA solution under specific conditions [111,112]. The coat did not critically affect the structure and architecture of the fibers but increased the fibers’ hydrophilicity and adhesiveness [111], resulting in a better attachment and spreading of cells together with an increase in proliferation rate. Moreover, in vivo experiments on full-thickness wound models, demonstrated that the PDA coating accelerated the healing process in comparison with uncoated membranes by providing a more adhesive surface and moist environment.

In a different approach, PDA-reduced graphene oxide (pGO) was incorporated into a SF/CS scaffold as a reinforcing and electroactive nanofiller [113]. Here, the pGO conferred to the dressing superior mechanical strength and stability in an aqueous environment. In addition, the material was provided with electroconductivity and antioxidant activity. The authors demonstrated that the electric stimulation provided through the dressing could regulate cellular behavior by promoting cell growth. In addition, the combined presence of pGO and PDA reduced the oxidative stress both in vitro and in vivo and decreased the inflammatory responses during wound healing.

Chen et al. have used PDA to immobilize the pineapple extract bromelain in electrospun poly(ε-caprolactone) (PCL) fibrous membrane [114]. Bromelain is a proteinase with a known therapeutic effect on wounds [115]. Indeed, it is able to mediate anti-inflammatory and anti-edematous effects and capable of hydrolyzing devitalized tissues, thus enhancing the wound healing process [116]. Electrospun fibers show a weak interaction with enzymes; in fact, bromelain-incorporated nanofibers have demonstrated reduced enzymatic activity and stability. Chen has found that the immobilization of bromelain via PDA onto PCL fibers markedly improved bromelain stability and supported cell attachment and proliferation [114]. The combined effect of bromelain and PDA also lend to in vitro antibacterial activity. Moreover, the obtained wound dressing accelerated the healing process while decreasing pro-inflammatory markers in a full-thickness wound model in rats [114].

**Figure 14:**
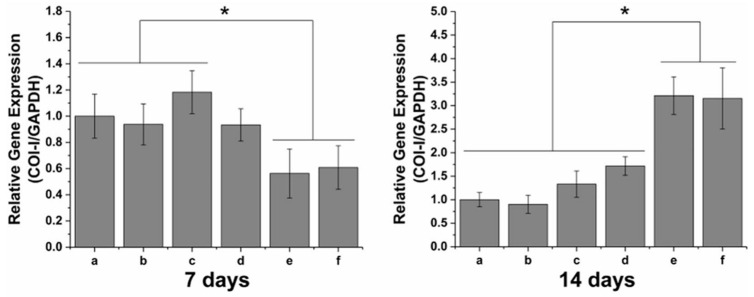
Quantitative polymerase chain reaction (qPCR) analysis of type 1 collagen expression by mouse embryonic fibroblast cells cultured on (**a**) poly(lactic-co-glycolic acid) (PLGA), (**b**) PonG1@PLGA, (**c**) DOPA-PonG1@PLGA, (**d**) bFGF@PLGA, (**e**) DOPA-bFGF@PLGA, and (**f**) DOPA-bFGF/DOPA-PonG1@PLGA for 7 and 14 days. * p < 0.05. Reproduced with permission from [117]. Copyright Elsevier, 2020.

Zhan et al. have exploited the DOPA to functionalize poly(lactic-co-glycolic acid) (PLGA) electrospun nanofibrous films with basic fibroblast growth factor (bFGF) and ponericin G1 (PonG1) [117]. To this aim, DOPA molecules were introduced to bFGF and PonG1 via recombinant DNA technology and subsequently applied to the PLGA films. Due to the presence of cresol moieties, DOPA sharply increased the binding affinity of recombinant bFGF and PonG1 for PLGA and allowed the sustained release of both bioactive factors over time. In addition, DOPA dramatically improved the hydrophilicity of PLGA membranes without affecting their mechanical properties. Moreover, the DOPA-PonG1@PLGA nanofibrous film demonstrated long-term antibacterial activity compared to both bare PLGA and PonG1@PLGA. In vitro experiments proved that DOPA-modified proteins increased cell growth and proliferation and could promote collagen deposition and ECM formation (Figure 14). Finally, in vivo wound healing evaluation revealed that DOPA-modified proteins, and, in particular, the combined DOPA-bFGF/DOPA-PonG1@PLGA formulation, accelerated the wound closure. Table 2 summarizes melanins and melanin-like materials for wound healing and the in vitro/in vivo models they have been tested in.

### 3.2. Bone and Cartilage Tissue Engineering

Bone grafting is the second most frequent tissue transplantation worldwide, with over two million procedures performed every year [118]. To date, autografts are the gold standard for bone healing and remodeling, with both structural and immunological compatibility [119]. Allo- and xenografts are also common in clinical; nonetheless, human/animal-derived tissue grafts present some significant limitations, including limited supply and high financial costs. A variety of synthetic bone substitutes, including metals, ceramics, and polymers, have been proposed to overcome the limits of natural bone grafts. Critical requirements for these materials include osteoconductivity, osteoinductivity, and osteointegration ability [120]. Natural bone is a complex system with a well-defined hierarchical organization of apatite minerals and fibrillar proteins [121]; bone scaffolds should closely mimic bone architecture and biomineralization to enhance cell recruitment, proliferation, and differentiation, finally allowing bone morphogenesis. Similar considerations are valid for cartilage implants. In tissue engineering, surface modifications are a common strategy to adjust the material’s interface and favor cell–scaffold interactions. Synthetic bone scaffolds often have a hydrophobic and bioinert nature, inadequate for cell activities. Melanin-like polymers are an easy and cheap way to improve the physicochemical features of bone substitutes to stimulate stem cell and osteoblast functions [122,123]. In addition, such polymers have also been demonstrated to promote the biomineralization process. Indeed, Ryu and collaborators have proposed a universal route to functionalize organic and inorganic materials by using PDA [124]. Such a route allows integrating hydroxyapatite (HA) in several different materials, including ceramics, metals, and polymers. Indeed, the free catechol and ammine moieties of PDA offer a binding site for calcium ions, which serve themselves as an anchor point for phosphate ions, leading to the formation of calcium phosphate layers in the physiological environment.

#### 3.2.1. Fibrous Scaffolds

**Figure 15:**
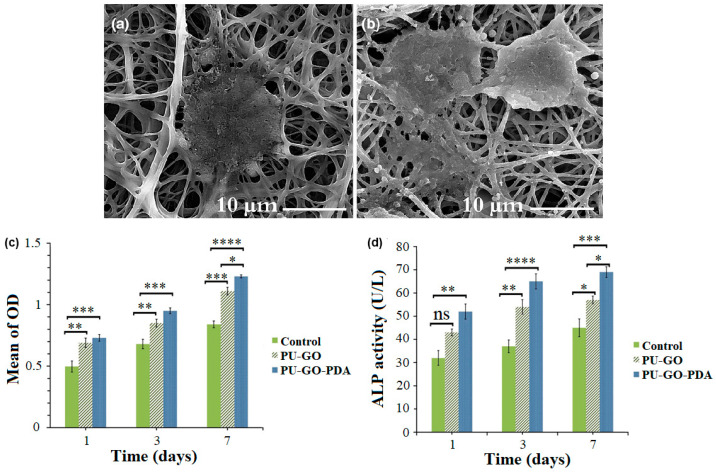
Field emission scanning electron microscopy micrographs of MG-63 (human osteosarcoma) cells attachment on GO-PDA (**a**) and PU-GO-PDA (**b**) fibrous scaffolds after 24  h. (**c**) Cell proliferation of MG-63 cells cultured in the presence and the absence of PDA, for 1, 3, and 7 days (* *p*  <  0.05, ** *p*  < 0.005, *** p < 0.0005, **** p < 0.00005). (**d**) alkaline phosphatase (ALP) activity of MG-63 cultured in the presence or absence of PDA, for 1, 3, and 7 days (* *p* <  0.05, ** *p*  <  0.005, *** p < 0.0005, **** p < 0.00005). Reproduced with permission from [130]. Copyright John Wiley and Sons, 2019.

Fibrous scaffolds are gaining increasing attention in bone tissue engineering because of their morphology, able to mimic the architecture of ECM and stem cell niche closely; additionally, the high surface to volume ratio provides a wide area for stem cell attachment and differentiation. As discussed above, despite their attractive morphological properties, these scaffolds lack biological recognition. Several in vitro studies have demonstrated that PDA and DOPA coatings are effective in promoting stem cells and osteoblasts adhesion and growth on different fibrous scaffolds [125,126,127,128,129]. For example, a recent study has investigated the behavior of osteoblast-like cells exposed to a PDA-coated polyurethane (PU)-GO electrospun scaffold [130]. PDA deposition did not affect the structure of the PU-GO scaffold but significantly improved its hydrophilicity. In vitro studies regarding the scaffolds’ bioactivity revealed that the presence of PDA increased the deposition of HA in physiological conditions. Moreover, when incubated with human osteoblast-like cells, PU-GO-PDA scaffolds were characterized by higher density, spreading, and proliferation rate compared to bare PU-GO scaffolds. Finally, the alkaline phosphatase (ALP) activity in PU-GO-PDA seeded cells was significantly higher than PU-GO samples, indicating that PU-GO-DA scaffolds possess osteogenic ability (Figure 15).

PDA was also found able to induce the osteogenic differentiation of dental derived stem cells [131]. Indeed, Hasani-Sadrabadi et al. have found that the presence of a PDA layer on PCL membranes directed the fate of gingival, periodontal ligament, and bone marrow-derived stem cells toward an osteogenic phenotype, as indicated by the expression of early osteogenic markers [131]. In vivo results in a periodontal defect model demonstrated higher levels of bone gain on coated membranes compared to bare PCL and sham groups. Notably, PDA nanoparticles have been proposed recently as an effective local antioxidant treatment in periodontal disease [132]. Thus, such findings support the possible use of PDA-coated membranes for guided periodontal tissue regeneration [131].

PDA degradation products have been found able to suppress inflammation in vitro [133]. Despite this, some authors hypothesized possible adverse effects deriving from PDA debrides for long-term implantations [134]. In view of this, Deng et al. have designed a PDA-incorporated PCL (PDA/PCL) hybrid fibrous membrane [134]. The membrane was obtained by coelectrospinning PDA nanoparticles and PCL, aiming to avoid the release of PDA debrides. Compared to the pristine membrane, the presence of PDA nanoparticles in the PDA/PCL membrane dramatically increased, in a dose-dependent manner, the in vitro deposition of HA. In addition, human mesenchymal stem cells demonstrated better spreading and proliferation, as well as a higher osteogenic differentiation. In vivo data obtained in a mouse skull defect model proved that the PDA/PCL membranes could accelerate bone remodeling compared to the pure PCL scaffold. 

#### 3.2.2. 3D Printed Scaffolds

**Figure 16:**
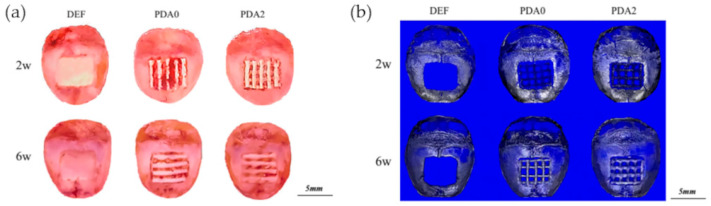
Gross specimens (**a**) and micro-CT-reconstructed images (**b**) of experimental animals at 2 weeks and 6 weeks after bare or PDA-coated scaffold-implantation surgery. Reproduced with permission from [144]. Copyright Multidisciplinary Digital Publishing Institute (MDPI), 2019.

Conventional scaffold processing, such as solvent casting, electrospinning, or phase separation, have been proven effective to fabricate bone scaffolds. Nonetheless, such processes are not only complex and require the use of toxic chemicals [135] but also allow for limited control of the scaffold geometry at microscopic and macroscopic levels [136]. Moreover, because of their limited mechanical properties, the application of such scaffolds is restrained to nonweight-bearing bone defects [136]. 3D printing has great potential in bone tissue engineering. First, due to computer-aided design, the manufacturing process is fast, simple, and does not need organic solvents. In addition, 3D printing enables to create scaffolds with customized shapes and tailored macro/microarchitecture [137]. Typically, 3D-printed (3DP) scaffolds are made of synthetic polymers or metals; thus, they present poor surface properties typical of inert materials. The researchers have made several attempts to modify the surface chemistry of 3DP scaffolds [138,139,140,141] and have found a convenient route in melanin-like polymers. Several in vitro studies reported the successful use of DOPA and PDA to enhance the bioactivity of 3DP scaffolds and to efficiently immobilize proteins, growth factors, or active molecules, such as type I collagen and bone morphogenic protein, on the implant surface [135,142,143]. Several studies have investigated the effect of modified 3DP scaffolds in preclinical animal models. For example, Xu et al. have investigated the effects of PDA coating on the osteointegration of 3DP PLGA/β-tricalcium phosphate (TCP) scaffolds [144]. The results indicated that the PDA coating significantly increased the attachment and spreading of pre-osteogenic cells in vitro. Proliferation and expression of osteogenic markers were significantly enhanced, too, with the better results obtained for the scaffold with higher PDA concentration (2 mg/mL, PDA2). The evaluation of osteogenesis in vivo revealed that the PDA-coated scaffold had a higher osteogenic potential; indeed, significant new bone formation was observed from week 2 to week 6, with the highest density observed for PDA2 groups (Figure 16). Similar results in terms of in vivo osteogenesis have been obtained employing PDA-coated titanium implants [145].

PDA has also been exploited as a reducing platform to grow GNPs on a 3DP PCL scaffolds [146]. GNPs can promote osteogenic differentiation [147]; thus, the authors have attempted to create a GNPs-coated scaffold for bone remodeling without using any toxic agent. PDA effectively enhanced the formation of GNPs on the scaffold surface, compared to bare PCL scaffold, resulting in good osteogenic activity in human adipose stem cells.

Table 3 summarizes melanin-like materials for bone regeneration and the in vitro/in vivo models they have been tested in.

### 3.3. Nerve and Muscular Tissue Engineering

The conductive properties of melanin, as well as its self-healing ability, have suggested the potential application of melanin and melanin-like materials for neural tissue engineering. Following severe nerve injury, the repair process is often incomplete, with low functional recovery [148]. Under these circumstances, autologous transplantation represents the first-choice treatment. Nonetheless, such a strategy presents numerous limitations, including lack of harvesting sites, rejections, and high costs for multiple surgeries [148]. Polymeric biomaterials present several advantages over biological grafts. First, polymers are usually economical, easy to be manufactured, and available in large amounts. In addition, polymer structure can be adjusted on specific requirements, to control biodegradation, favor cell attachment, and mimic the mechanical strength and architecture of the tissue that should be replaced. Nonetheless, most of the available biomaterials do not meet the electrochemical properties of neural tissues, thus impeding optimal biointegration. Melanins represent a convenient solution to improve the interaction between neural cells and synthetic devices. In 2009, Bettinger and collaborators characterized a film of pure synthetic melanin for neural tissue engineering applications [149]. The melanin film, produced via spin-coating, showed semiconductive properties in a fully hydrated state, as expected in physiological conditions. The authors have demonstrated that both rat Schwann and PC12 (a pheochromocytoma cell line) cells showed a more activated phenotype when cultured on melanin films compared to collagen-coated or uncoated supports. Moreover, in vivo experiments demonstrated that the foreign response of sciatic nerve and surrounding tissues to synthetic melanin was qualitatively similar to those induced by silicone, a material commonly used for peripheral nerve reconstruction. Nonetheless, compared to silicon, melanin presented a faster degradation rate, being almost wholly degraded 8 weeks after implantation.

More recently, on the path of melanin-modified materials, melanin has been employed to endow different materials with cytoaffinity and conductivity, to make them suitable for neural tissue engineering [150]. Several works report the successful use of PDA coating to enhance nerve cell attachment and organization on nerve conduits [151,152,153,154]. Besides, melanins can be exploited to immobilize neurotrophic factors on synthetic nerve grafts, generating physical and biochemical signals able to modulate neural cell proliferation and differentiation. For example, Pan et al. have adsorbed the nerve growth factor (NGF) onto a porous PLGA scaffold via PDA surface modification [155]. PDA-treated scaffolds sustained the proliferation and differentiation of neuronal stem cells to neurons. Such effect was further improved by the presence of NGF, which also induced a pronounced elongation of neuron axons. The proregenerative effects of the scaffold were then confirmed in a rat spinal cord transection model. Here, for PDA-PLGA/NGF-treated animals, a restoration of motor functions and a reduction of the spinal cord tissue defect were observed. Although NGF plays a critical role in the regenerative effects, PDA appears to be essential to enhance the adsorption capacity of PLGA for NGF and to allow its sustained release.

**Figure 17:**
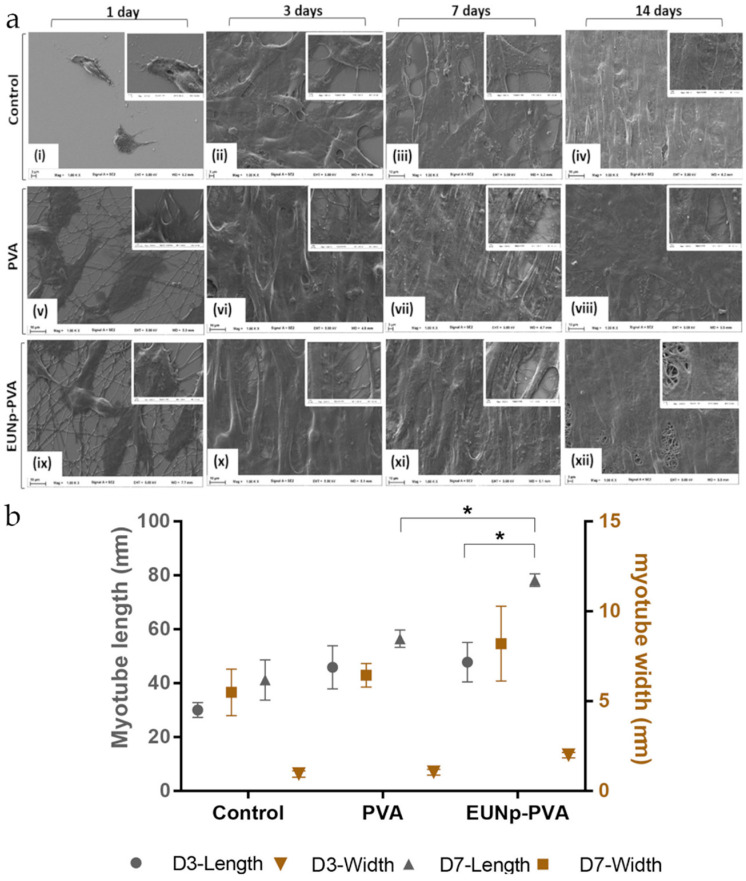
(**a**) Myogenic differentiation of C2C12 on nanofibrous scaffolds. SEM micrographs of myoblasts represent the control (**i**), polyvinyl alcohol (PVA) (**v**), and eumelanin nanoparticles-PVA (**ix**) at day 1; control (**ii**), PVA (**vi**), and eumelanin nanoparticles-PVA (**x**) at day 3; control (**iii**), PVA (**vii**), and eumelanin nanoparticles-PVA (**xi**) at day 7; and control (**iv**), PVA (**viii**), and eumelanin nanoparticles-PVA (**xii**) at day 14. (**b**) Quantification of myotube formation during differentiation of C2C12 on PVA, and eumelanin nanoparticles-PVA nanofibrous scaffolds and respective controls at day 3 and day 7. (* *p* < 0.05). Reproduced with permission from [158]. Copyright American Chemical Society, 2018.

Preliminary studies have demonstrated that melanin composite SF scaffolds support the differentiation and the organization of myoblast into myotubes, suggesting a possible application of melanin composite materials in skeletal muscle regenerative approaches [88,156]. In addition, the conductive properties of melanins can support the growth of cardiac tissue and assist in restoring the conduction of electric signals [157]. On this path, eumelanin nanoparticles from Sepia ink have been incorporated in a PVA nanofibrous membrane to obtain an electroconductive scaffold for skeletal muscle tissue engineering [158]. Eumelanin nanoparticles positively influenced mouse myoblasts’ behavior, inducing both proliferation and differentiation, and the organization into myotube-like structures (Figure 17).

In addition, Jing et al. have combined PDA and GO to produce a multifunctional CS hydrogel [159]. The hydrogel demonstrated the ability to enhance the viability and proliferation of human embryonic stem cell-derived fibroblasts and cardiomyocytes. Interestingly, the cardiomyocytes showed a spontaneous beating activity, which was two times higher for the samples seeded on the composite hydrogel than on control tissue culture plates. Table 4 summarizes melanin and melanin-like materials for neural tissue engineering and the in vitro/in vivo models they have been tested in.

## 4. Conclusions

A growing body of literature reports the successful use of synthetic melanin-like materials and their derivatives as doping/coating materials for several types of dressings and scaffolds for tissue regeneration. These multifunctional polymers can improve the physical, chemical, and mechanical characteristics of a wide variety of materials for skin, bone, and neural tissue repair. Moreover, they allow for the immobilization and controlled release of therapeutic agents or biomolecules (growth factors, protein, or cytokines) on materials’ surfaces. In addition, because of their prominent photothermal properties, melanins could be exploited to obtain, through quite simple routes, smart dressings/scaffolds suitable for PTT. Photothermal stimuli could also work as a trigger to spatially or temporally control the release of immobilized molecules; thus, melanin-modified materials, in association with PTT, could have a high potential as both medications and drug delivery vehicles in tissue regeneration. Finally, the ionic/electronic conductivity suggests that melanin could be possibly used in electroactive tissue engineering, despite, currently, the reports in this specific field are limited and mostly confined at an in vitro level.

The use of melanins and their derivatives as coating materials has been extensively studied and, by now, appears as a well-established procedure to produce multifunctional materials for regenerative purposes. In contrast, nanostructured melanins, deeply investigated in other biomedical fields, are mostly unexplored in tissue regeneration. As reported, nanostructured melanins have good potential as theranostic agents. With proper designs, melanin nanoparticles could pave the way for smart materials suitable for both tissue engineering and bioimaging applications. Such devices could restore tissue structure and functions and allow for monitoring healing progression with noninvasive techniques, such as PAI or MRI. Thus, focusing the research efforts on the development of nanostructured systems could represent a step forward in the application of melanins in tissue engineering.

## Figures and Tables

**Table 1:** Summary of melanins and melanin-like hybrid materials.

Materials	Organic Component	Functionality	Application	Ref.
SiO_2_ shell coated/gadolinium-chelated melanin nanoparticles	Synthetic melanin from DA	Heat transduction efficiency	In vivo bimodal MRI/fluorescent imaging	[50]
Ag/melanin/SiO_2_ hybrid nanostructures	Synthetic melanin from DHICA	Photoacoustic properties under long irradiation	PAI	[51]
Melanin/TiO_2_ hybrid nanostructures	Synthetic melanin from DHICA	Reactive oxygen species (ROS) production and bacteria killing	Antimicrobial agents	[22,45,46]
Metal ion-loaded synthetic melanin	Synthetic melanin from DA	NIR light absorption	NIR imaging	[52]
Metal-bound natural melanin particles	Natural melanin	Enzyme mimicking activity	ROS scavengers and in anti-inflammatory agents	[53]
PEGylated-ions doped melanin nanoparticle	Synthetic melanin from DA	Theranostic properties	In vitro and in vivo MRI/computed tomography (CT) imaging agents	[55]
Metal ion-synthetic melanin nanoparticles	Synthetic melanin from DA	Metal ion chelating/light absorption ability and photothermal effects	NIR imaging	[52,54,55,56,69,70,87]
Gd^3+^-loading melanin dots	Commercial melanin	Higher T1 relaxivity	PAI	[44,56]
PDA gels/nanoparticles	PDA	Photoprotection	Sunscreen	[58,60]
Melanin-loaded nanovesicles	Natural melanin	High photothermal conversion	NIR imaging/PTT	[68]
Hydrogel scaffolds	Grape extracts oligomeric proanthocyanidins	Photothermal conversion	Melanoma therapy and wound healing	[64]
Polymer based-melanin hydrogels	Natural/synthetic melanin	Biocompatibility	Biomedical application	[65,66,67,68]
Gold core-melanin shell nanoparticles	Synthetic melanin from DA	Enhanced photoacoustic conversion	PAI	[71]
Metal carbonates-melanin nanoparticles	PDA	Metal ion chelating/light absorption ability and photothermal effects	MRI/PTT theranostic agents	[72,73]
Melanin-nanoparticles incorporated agar-based composites	Natural melanin from Sepia ink	ROS scavenging	Films with antioxidant activity	[74]
CS/melanin-like nanocomposites	Synthetic melanin from DA	ROS scavenging (antioxidants)	Films for food packaging and biomedical packaging	[75]
Melanin-like polymer complex	Natural melanins from olive mill wastewaters	High radical scavenging activity	Antioxidant agents	[76]
Silk/melanin nanofibrous scaffolds	Commercial melanin	Antioxidant and radical scavenging properties	Nerve tissue engineering	[88]
Hybrid silver-loaded/melanin spheres.	Synthetic melanin from DA	ROS generation	Antibacterial agents	[78]
Bioinspired coatings	Mussel melanin	Adhesive properties	Active biointerfaces	[85]
Poly(norepinephrine) coatings	Norepinephrine polymerization	Biocompatibility and adhesive properties	Human neural stem cells adhesion	[35]
DA modified PANI hybrids	Synthetic melanin from DA	Adhesion, dispersibility, and biocompatibility	Conductive platforms in tissue engineering	[86]

**Table 2:** Summary of melanins/melanin-like materials for wound healing.

Matrix	Melanin/Melanin-Like Material	Additives	Experimental Model(s)		Ref.
			In Vitro	In Vivo	
PAM, nanoclay	PDA	EGF	NIH-3T3 fibroblasts (mouse)	Full-thickness skin excision (rat)	[94]
PEG	PDA	PUE	Dental pulp stem cells, periodontal ligament stem cells	Full-thickness skin excision (rat)	[95]
CS/SF	PDA	–	NIH-3T3 fibroblasts (mouse)	Full-thickness skin excision (rat)	[101]
Gelatin, carbon nanotubes	PDA	–	L929 fibroblasts (mouse)	Bleeding liver, full-thickness skin excision (mouse)	[102]
Hyaluronic acid, GO	PDA	–	L929 fibroblasts (mouse)	Bleeding liver, full-thickness skin excision (mouse)	[97]
Nanocellulose	PDA	TH	–	Full-thickness skin excision (rat)	[104]
G-C	PDA	Cyp	Normal lung cells (AT-II) (human)	*S. aureus*-infection model, full-thickness skin excision (mouse)	[105]
PNIPAM	PDA	–	NIH-3T3 fibroblasts (mouse)	Full-thickness skin excision (rat)	[96]
PANI, PVA	PDA	Silver	–	*S. aureus-* and *E. coli*-infected diabetic wound (rat)	[98]
PAM, GO	PDA	–	–	Full-thickness skin excision (mouse)	[108]
Gellan gum	Eumelanin nanoparticles (*Sepia officinalis*)	–	Primary keratinocytes (human), C3H/a fibroblast-like cells (mouse)	Subcutaneous implantation (mouse)	[99]
SF	PDA	-	L929 fibroblasts (mouse)	Full-thickness skin excision (mouse)	[111]
SF	PDA	-	Mesenchymal stem cells (rat)	Full-thickness skin excision (rat)	[112]
SF, CS, GO	PDA	-	C2C12 myoblasts (mouse)	Full-thickness skin excision (rat)	[113]
PCL	PDA	Bromelain	L929 fibroblasts (mouse)	Full-thickness skin excision (rat)	[114]
PLGA	DOPA	bFGF PonG1	BALB/c 3T3 fibroblasts (mouse embryonic)	Full-thickness skin excision (rat)	[117]

**Table 3:** Summary of melanin-like materials for bone tissue engineering.

Matrix	Melanin/Melanin-Like Material	Additives	Experimental Model(s)		Ref.
			In Vitro	In Vivo	
PCL, gelatin	PDA	–	Adipose-derived stem cells (mouse)	–	[125]
PLA	PDA	–	Adipose-derived stem cells (human)	–	[126]
PU cellulose nanofibers	PDA	–	MC3T3-E1 embryonic osteoblasts (mouse)	–	[127]
PANI poly(d,l-lactide)	PDA	–	MC3T3-E1 embryonic osteoblasts (mouse)	–	[128]
PCL	PDA	–	Mesenchymal stem cells (human)	–	[129]
PU, GO	PDA		MG-63 osteosarcoma cells (human)	–	[130]
PCL	PDA	–	Dental-derived stem cells (human)	Periodontal defect (rat)	[131]
PCL	PDA	–	Mesenchymal stem cells (human)	Skull defect (mouse)	[134]
PCL	PDA	recombinant human bone morphogenetic protein-2	MC3T3-E1 embryonic osteoblasts (mouse)	–	[135]
PLA, type I collagen	PDA	–	Bone marrow stem cells (pig)		[142]
Poly(lactide)	PDA	quercetin	MC3T3-E1 embryonic osteoblasts (mouse)	–	[143]
PLGA/TCP	PDA	–	MC3T3-E1 embryonic osteoblasts (mouse)	Critical size skull defect (mouse)	[144]
porous titanium	PDA		–	Femoral condyle defect (rabbit)	[145]
PCL	PDA	GNPs	Adipose-derived stem cells (human)	Skull defect (rabbit)	[146]

**Table 4:** Summary of melanin-doped materials for neural tissue engineering.

Matrix	Melanin/Melanin-Like Material	Additives	Experimental Model(s)		Ref.
			In Vitro	In Vivo	
–	Synthetic melanin film	–	Schwann cells (rat)	Implantation on top of the sciatic nerve (rat)	[149]
Glass, platinum, indium tin oxide	PDA	–	Hippocampal neurons (rat)	–	[150]
PU, decellularized ECM	PDA	–	L929 fibroblasts (mouse), Schwann cells (human)	–	[151]
Polystyrene	PDA	–	PC12 pheochromocytoma cells (rat), adipose-derived stem cells (human)	–	[152]
PCL, gold	PDA	–	Bone marrow stem cells, Schwann cells (rat)	Sciatic nerve dissection (rat)	[153]
PCL, graphene	PDA	–	Schwann cells (rat)	Sciatic nerve dissection (rat)	[154]
PLGA	PDA	NGF	Neural stem cells (mouse)	Spinal cord injury (rat)	[155]
SF	Synthetic melanin	–	SH-SY5Y neuroblastoma cells (human)	–	[88]
SF	Synthetic melanin	–	C2C12 myoblast cells (mouse)	–	[156]
poly(L-lactide-co-ε-caprolactone), gelatin	Synthetic melanin	–	Cardiac myocytes (human)	–	[157]
PVA	Eumelanin nanoparticles from Sepia ink	–	C2C12 myoblast cells (mouse)	–	[158]
CS, GO	PDA	–	HEF1 fibroblast cells, cardiomyocytes (human)	–	[159]

## Abbreviations

3DP	3D printed
ALP	alkaline phosphatase
bFGF	basic fibroblast growth factor
CS	Chitosan
CT	computed tomography
Cyp	Ciprofloxacin
DA	Dopamine
DHI	5,6-dihydroxyindole
DHICA	5,6-dihydroxyindole-2-carboxylic acid
EDC	1-ethyl-3-(3-dimethylaminopropyl)carbodiimide
EGF	epidermal growth factor
G-C	glycol-chitosan
GO	graphene oxide
hKCs	human keratinocytes
L-DOPA	L-3,4-dihydroxyphenylalanine
LMCT	ligand to metal charge transfer
MRI	magnetic resonance imaging
NGF	nerve growth factor
NIR	near-infrared
PAI	photoacoustic imaging
PAM	Polyacrylamide
PANI	Polyaniline
PCL	poly(e-caprolactone)
PDA	Polydopamine
pDHN	poly-1,8-dihydroxynaphthalene
PEG	poly(ethylene glycol)
PET	positron emission tomography
pGO	polydopamine-reduced graphene oxide
PLA	poly(lactic acid)
PLG	poly(L-lactide-co-glycolide)
PLGA	poly(lactic-co-glycolic acid)
PMPDA NPs	PEGylated Mn2+−PDA nanoparticles
PNIPAM	poly(N-isopropylacrylamide-co-acrylamide)
PonG1	ponericin G1
PTT	photothermal therapy
PU	Polyurethane
PUE	Puerarin
PVA	polyvinyl alcohol
qPCR	quantitative polymerase chain reaction
RONS	reactive oxygen and nitrogen species
ROS	reactive oxygen species
SEM	scanning electron micrographs
SF	silk fibroin
SMNP	synthetic melanin nanoparticles
SNpc	substantia nigra pars compacta
TCP	β-tricalcium phosphate
TEM	transmission electron microscopy
TH	tetracycline hydrochloride
UV	Ultraviolet
